# Empowering Children With Down Syndrome by Enhancing Emergency Preparedness Through Serious Games: Quasi-Experimental Study With a Between-Group Design

**DOI:** 10.2196/73690

**Published:** 2025-10-17

**Authors:** Samaa M Shohieb, Suzan Hassan Bakhit, Ensaf Mohammed, Abdelghafar M Elhady

**Affiliations:** 1 Faculty of Computers and Information Mansoura University Mansoura Egypt; 2 Department of Computer Engineering and Information College of Engineering-Wadi Addawasir Prince Sattam Bin Abdulaziz University Reyad Saudi Arabia; 3 General Administration of the University Mansoura University Mansoura Egypt

**Keywords:** crisis management, Down syndrome, dynamic difficulty adjustment, game-based learning, game-based rehabilitation, machine learning, serious games

## Abstract

**Background:**

Children with Down syndrome (DS) often experience cognitive and adaptive challenges that affect their ability to acquire and retain critical life skills, including those needed for effective response during emergencies. Traditional training methods used to prepare children for crises are frequently static, noninteractive, and insufficiently tailored to the unique learning profiles of children with DS. These limitations contribute to reduced engagement, poor knowledge retention, and inadequate real-world preparedness. Recent advancements in game-based learning, particularly serious games, have demonstrated potential for enhancing education and skill development among individuals with cognitive impairments.

**Objective:**

This study aimed to design, implement, and evaluate Risk Resist, an adaptive serious game developed to improve emergency preparedness in children with DS. The game incorporates a dynamic difficulty adjustment algorithm that personalizes the learning experience by dynamically modifying game difficulty based on real-time behavioral performance metrics. The study also assessed whether this adaptive game-based learning approach leads to superior learning gains and engagement compared to conventional teacher-led training.

**Methods:**

A quasi-experimental, between-group design was used with 18 children diagnosed with DS, aged 8 to 12 years. Participants were randomly assigned to either an experimental group (n=9), which played Risk Resist, or a control group (n=9), which received traditional instruction on emergency scenarios. Learning outcomes were assessed using pre- and postintervention knowledge tests composed of 5 emergency-related questions. Engagement levels were measured through a structured 5-point Likert scale questionnaire completed by observing teachers. The game used a machine learning–driven dynamic difficulty adjustment model, specifically a Random Forest Regressor, which adjusted difficulty in response to individual performance indicators such as success rate, response time, and behavioral patterns during gameplay.

**Results:**

The experimental group achieved significantly higher learning gains (mean 3.6, SD 0.5) than the control group (mean 2.0, SD 0.5; *P*<.001). Engagement levels were also significantly greater in the game-based group (mean 4.54, SD 0.4) compared to the control group (mean 4.01, SD 0.32; *P*=.002). A strong positive correlation was identified between engagement and learning gain (*r*=0.85; *P*<.001), indicating that higher engagement contributed directly to improved knowledge acquisition.

**Conclusions:**

The results support the effectiveness of Risk Resist in enhancing both engagement and learning outcomes for children with DS. The integration of adaptive difficulty algorithms provides a personalized, responsive learning experience, positioning serious games as a viable and impactful tool for emergency preparedness training in special education settings.

## Introduction

Children with Down syndrome (DS) represent a significant portion of the global population, with approximately 1 in every 700 children born each year affected by this chromosomal abnormality [1]. As the most common chromosomal condition, DS presents unique challenges in cognitive development and skill acquisition, necessitating specialized educational strategies to optimize learning outcomes [2].

### Problem Statement and the Significance of the Study

Children with DS face significant cognitive and adaptive challenges that affect their ability to respond to emergencies effectively. Difficulties in processing complex instructions, recalling learned responses, and making rapid safety decisions increase their vulnerability during crises [3]. Many struggle to interpret danger cues, follow emergency protocols, and apply prior knowledge under stressful conditions, making traditional preparedness training methods insufficient [4].

Despite efforts to teach crisis response, conventional teacher-led instruction often fails to engage children with DS effectively, as these methods lack interactive and adaptive learning mechanisms [5]. The limitations of static training approaches reduce knowledge retention and do not accommodate the individualized learning needs of children with DS. Consequently, children may remain underprepared for real-world emergencies due to the absence of engagement-driven learning models [6,7].

To address these gaps, this study introduces Risk Resist, a game-based learning (GBL) tool designed to provide personalized, adaptive emergency training through interactive gameplay and real-time feedback. In addition to addressing the immediate learning needs of children with DS, the development of adaptive serious games like Risk Resist has broader implications for educational policy and curriculum design in special education. By providing personalized, engaging, and scalable learning tools, serious games can complement traditional instructional methods and support inclusive education mandates. This aligns with growing international calls for leveraging technology to enhance educational accessibility [8], equity, and lifelong learning opportunities for individuals with cognitive disabilities. By incorporating dynamic difficulty adjustment (DDA) and engagement-driven mechanics, Risk Resist enhances learning retention and supports cognitive accessibility for children with DS, ensuring a safe and effective learning experience.

Emergency preparedness is essential for ensuring the safety and well-being of individuals in crises. However, children with DS face unique cognitive challenges that impair their ability to process emergency information, recall procedures, and react appropriately under stress. Traditional training methods, such as teacher-led instruction or passive learning approaches, fail to engage these children effectively and lack the adaptive mechanisms necessary for personalized crisis management training. These limitations hinder learning retention, reduce motivation, and fail to equip children with practical, real-world emergency response skills [3,4,6].

GBL has demonstrated significant benefits for engagement, knowledge retention, and skill acquisition, particularly for individuals with cognitive disabilities [7]. However, conventional static GBL designs do not automatically adjust difficulty based on individual learning progression, making them less effective for children with DS, whose attention span, cognitive processing speed, and learning abilities vary significantly.

To address these challenges, this study introduces Risk Resist, an adaptive serious game that incorporates DDA to personalize learning and optimize engagement levels. DDA dynamically modifies challenge levels based on player performance metrics, ensuring that learning remains accessible, motivating, and appropriately challenging. By using machine learning techniques, the game adjusts difficulty in real time, preventing frustration from overly difficult scenarios while ensuring continued cognitive engagement and learning progression.

The integration of adaptive mechanics in serious gaming for crisis preparedness represents an innovative, evidence-based approach to enhancing emergency response skills in children with DS. Risk Resist bridges the gap between static training methods and real-world applicability, ensuring effective knowledge transfer while maintaining high levels of engagement and accessibility.

### Literature Review

#### Overview

To identify relevant studies on serious games designed for children with DS, a literature search was conducted using multiple academic databases, including PubMed, Scopus, IEEE Xplore, and Web of Science. The search included publications up to 2024, using combinations of keywords such as “serious games,” “Down syndrome,” “educational games,” “cognitive development,” and “assistive technologies.” Studies were included if they (1) focused on the development, evaluation, or application of serious games for individuals with DS; (2) addressed cognitive, motor, language, or daily living skill development; and (3) were peer-reviewed journal papers or conference proceedings. Exclusion criteria included studies not specifically targeting individuals with DS, nonpeer-reviewed literature, and papers lacking empirical data or detailed descriptions of the game design. This selection process ensured that the reviewed literature was directly relevant to the target population and aligned with the scope of this research.

#### Serious Games for DS

The use of serious games has been extensively explored as a means to provide individuals with DS essential daily concepts and training for various life situations. For instance, “Junk-Food Destroyer,” a video game developed by Hatzigiannakoglou [9], is designed to help teenagers with DS understand the importance of healthy eating. Similarly, “EnCity,” developed specifically for individuals with DS by Bourazeri et al [10], includes a variety of real-life skills such as meal preparation and bill payment, encouraging players to engage without fear of failure.

Brandão et al [11] proposed a video game aimed at aiding preschool children with DS in enhancing their cognitive development. They found that controlling a mouse promoted interaction between the game and the player. However, Macedo [12] noted that while players with DS enjoyed the game, they also experienced boredom and frustration in response to game stimuli. AKA New Media Inc developed online games to teach DS individuals aged 14 to 20 years to use the internet [13]. The authors suggested modifying these games to enhance engagement through auditory reinforcement, animated feedback, and immediate positive support.

González-Ferreras et al [14] conducted usability testing to demonstrate that DS games should adapt to players’ skills, incorporate age-appropriate graphics, provide clear instructions, and offer positive feedback. Research on the physical effects of video games on individuals with DS has shown that Wii games can improve sensorimotor skills [15], balance [16], and coordination [17]. Berg et al [17] observed significant improvements in a child’s balance and agility after 8 weeks of playing Nintendo Wii, although there were no bimanual coordination improvements [18]. Wuang et al [15] found that Nintendo Wii games motivated players to continue learning, attributing improvements to increased brain plasticity during gaming.

Whalen et al [19] used “TeachTown” to improve language and memory in 4-year-old children with DS, finding significant performance increases after 8 weeks. Lopez-Basterretxea et al [20] provided 3 money-related iPad apps to children with DS, revealing positive interactions and success in counting and recognizing money. Shalash et al [21] designed “No Limit,” an interactive game developed using Unity 3D and Kinect 360, to enhance the intellectual and physical abilities of children with DS, showing promising results during testing. The literature review of serious games for individuals with DS is displayed in Table 1, which includes “Research future work or gap” that summarizes the limitations, open issues, or suggested directions for future work identified in each referenced study. Including this column helps to highlight existing gaps in the literature that our own game seeks to address, such as limited real-life emergency preparedness interventions specifically tailored for children with DS.

**Table 1:** Serious games for children with Down syndrome (DS).

Author (year)	Game name	Purpose	Technique	Findings	Research future work or gap
Hatzigiannakoglou (2015) [9]	Junk-Food Destroyer	Teach teenagers with DS the importance of healthy eating	First-person shooting	Enhanced understanding of healthy eating	The game has not been applied to real users
Bourazeri et al (2017) [10]	EnCity	Teach real-life skills (eg, meal preparation and bill payment) to individuals with DS	Virtual reality serious game	Covered a broad range of daily skills	Continuous engagement with the end users (young people with DS)
Brandão et al (2010) [11]	Jecripe	Aid preschool children with DS in cognitive development	2D computer game	Promoted interaction via mouse control	Observe more users playing the game
Macedo (2015) [12]	Jecripe (evaluation)	Evaluate the previously proposed game	2D computer game	Proved the game’s efficiency for children with DS	Apply to more users
Kirijian et al (2007) [13]	Web Fun Central	Teach individuals with DS to use the internet	Online learning tools for DS	Individuals with DS use Web Fun Central across North America	Using the system for different cultural users
González-Ferreras et al (2017) [14]	N/A^a^	Improve voice skills, particularly prosody-related people with DS	Video game	Good level of acceptance by professionals	Include languages other than Spanish and redesign activities to train vocabulary, syntax, and phonetics
Wuang et al (2011) [15]	Wii game (not identified)	Improve motor skills using Nintendo Wii in children with DS aged 10-13 years	Virtual reality or Wii-Fit game	Significant improvements in balance	Apply to more users
Rahman and Rahman (2010) [16]	Wii game (not identified)	Measure the balance of children with DS	Virtual reality or Wii-Fit game	High improvement of equilibrium in the study group	N/A
Berg et al (2012) [17]	Wii game	Improve a child’s stability and postural control	Wii game	Need improvements in postural control and motor skills	N/A
Whalen et al (2006) [19]	TeachTown	Improve language and memory in children	Video computer game	Significant performance increases	Apply to more users
Lopez-Basterretxea et al (2014) [20]	iPad game	Count and recognize money for children with DS	OSI^b^ games on iPads	Positive interactions, successful money counting, and recognition	N/A
Shalash et al (2018) [21]	No Limit	Enhance motor and cognitive skills for young people	3D Unity game	Promising intellectual and physical enhancement	Add a wider range of coins and notes and design real-life activities like shopping at supermarkets

^a^N/A: not available.

^b^OSI: Open Systems Interconnection.

#### Crisis and Emergency Management Games

Several crisis and emergency management games have been developed to teach individuals how to handle various disaster scenarios. “StopDisasters” by the International Strategy for Disaster Reduction engages players in activities to manage a specified budget and mitigate potential disaster consequences. Players can choose specific natural disasters such as floods, tsunamis, wildfires, hurricanes, and earthquakes, aiming to allocate resources efficiently to reduce community vulnerability [22].

“Crisis of Nations” [23] is an international civics simulation, where players assume the role of a country and must collaborate with 3 other nations to resolve issues. Effective resource use is a key to winning the game, and a time limit ensures that the game does not continue indefinitely.

Babu et al [24] discussed learning from disasters through collaborative game-based disaster management training. In a multiplayer game, players face a simulated catastrophe with an incident management structure focusing on cooperation and communication. Students and academic members from Amrita University tested the game, showing that the proposed frameworks successfully provided fundamental postdisaster management knowledge to participants.

Shohieb [25] and Doenyas and Shohieb [26] proposed the “Crises and Disaster Management Game”, piloted by 12 students at a government primary school in Mansoura, Egypt. The results revealed that the Crises and Disaster Management Game efficiently provided basic crisis management knowledge to players.

Although these crisis management games have been beneficial for adults and young people, they are not always suitable for children with DS [27,28]. Additionally, these games are typically in English, which can be a barrier for those from Arabic cultures [29]. Consequently, “Risk Resist” was developed specifically for children with DS and features an English or Arabic interface.

### Objectives

The objectives of this study are 2-fold. This study introduces Risk Resist, an adaptive serious game designed to enhance emergency preparedness in children with DS. The primary aim is to evaluate the effectiveness of the game in improving both learning outcomes and engagement levels compared to traditional teacher-led training. Specifically, the study tests 2 hypotheses: (hypothesis 1) that children who use Risk Resist will demonstrate significantly greater learning gains than those receiving conventional instruction and (hypothesis 2) that Risk Resist will elicit significantly higher engagement levels. These hypotheses are examined through a controlled experimental design involving pre- and postintervention assessments and teacher-reported engagement metrics.

## Methods

### Overview

Effective learning for children with DS relies heavily on visual and interactive elements, as research has consistently demonstrated that illustrated content enhances comprehension and retention [30]. Educational approaches that integrate photos, animations, and interactive experiences have been shown to significantly improve learning engagement for individuals with DS, making game-based methodologies an ideal choice for emergency preparedness training.

Recognizing these cognitive needs, Risk Resist was specifically designed to ensure that children with DS could absorb and apply emergency response strategies effectively. Studies highlight that many children with DS struggle with basic life skills [6], which underscores the importance of reinforcing training concepts through adaptive gameplay mechanics. This principle aligns with Freina et al [31], who found that learning transfer—the ability to apply acquired knowledge across different environments—is significantly enhanced through immersive and interactive learning experiences.

### Risk Resist Game Design Process and Its Rationale

#### Concept Development and General Description of Risk Resist

Risk Resist is a GBL tool designed to teach crisis and emergency management in a safe, controlled environment, preventing real-world risks to children with DS. Through engaging gameplay, players can practice methodical crisis response, such as handling indoor fires, in a way that reinforces learning without exposure to danger. The game’s systemic framework allows for broad application across different types of emergencies, spanning both human-caused and ecological crises. The game is structured into two main categories: (1) human-caused crises includes electrical fires, first aid emergencies (such as fainting and bleeding), and road-crossing safety; and (2) natural catastrophes teaches essential survival strategies during disasters like earthquakes.

#### Learning Needs and Cognitive Accessibility

To ensure accessibility for children with DS, Risk Resist incorporates specialist recommendations and evidence-based learning theories [32-36]. The design specifically integrates:

- Visual learning: Vivid graphics, animations, and interactive storytelling engage players and reinforce emergency concepts.
- Repetition and feedback: Scenarios provide repeated exposure and immediate positive reinforcement, enhancing skill retention.
- Simplified instructions: Clear guidance, intuitive controls, and step-by-step instructions ensure accessibility and comprehension for children with DS.

### Game Mechanics and Adaptive Learning

**Figure 1:**
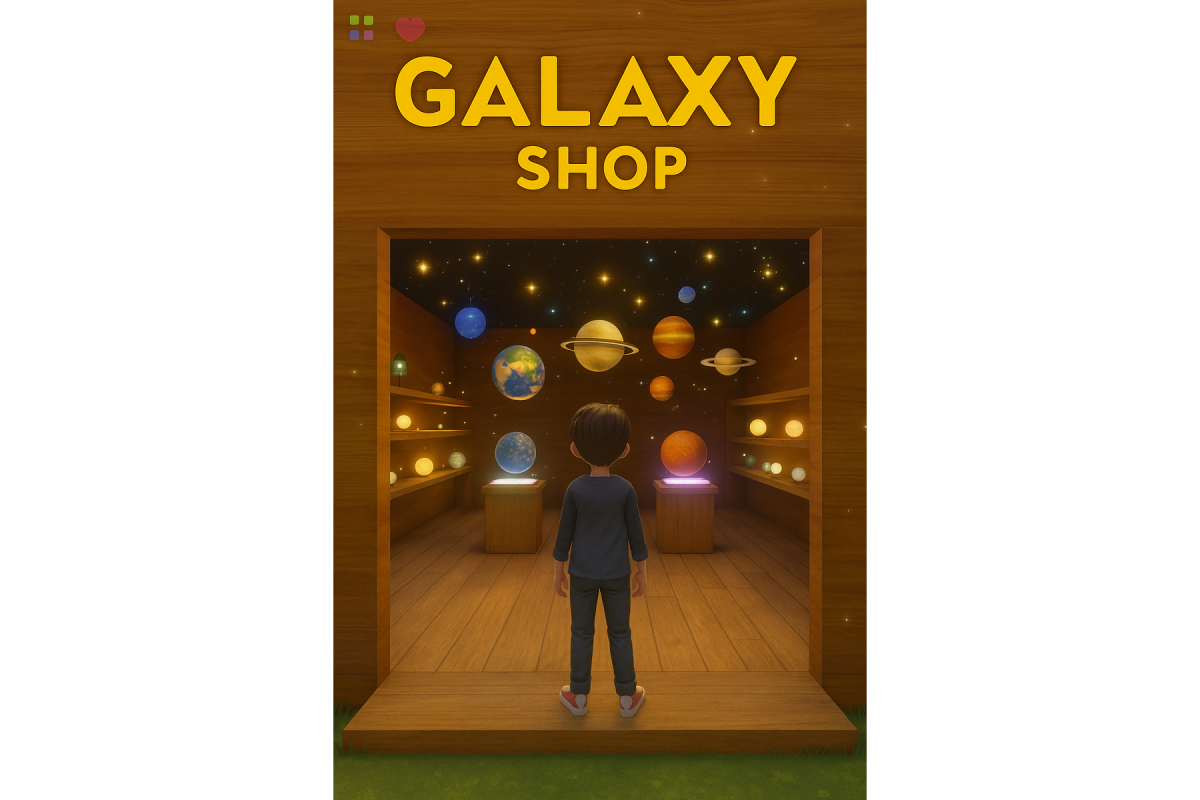
The Galaxy Shop.

The game’s homepage emphasizes peer support, fostering collaboration and engagement among students. The mechanics implement ongoing trials, ensuring players grasp key emergency responses through adaptive learning techniques essential for children with cognitive difficulties [7,32]. These features are seamlessly integrated within the Galaxy Shop, making them readily available for use within gameplay. The Galaxy Shop is a virtual store integrated within the game environment, where children can redeem points or rewards earned through gameplay for various customization options. These include avatar personalization, acquisition of accessories, and unlocking of bonus content, all of which are designed to maintain children’s interest and enhance their intrinsic motivation through gamification principles such as reward systems, autonomy, and personalization (Figure 1).

- Constructivist learning theory: Active learning where individuals acquire knowledge through experiential engagement rather than passive instruction. This aligns with Risk Resist’s interactive gameplay, which enables children to practice crisis management in a simulated environment, reinforcing problem-solving and decision-making skills [37,38].
- Skill transfer model: The concept of learning transfer refers to the ability to apply knowledge gained in one environment to real-world situations. Research indicates that learning transfer improves when activities mimic real-world challenges [30,31]. To promote skill transfer, Risk Resist integrates real-life scenarios, including fire emergencies, first-aid response, and road-crossing safety, ensuring that children develop applicable emergency skills beyond the gaming environment.
- Reinforcement theory: The game incorporates positive reinforcement by providing instant feedback, reward mechanisms, and gradual difficulty progression. According to Skinner’s reinforcement theory [39], positive reinforcement (praise, progress tracking, and visual rewards) enhances motivation and learning retention.
- Prototype creation and iterative design are described in detail in Multimedia Appendix 1.

### Technical Implementation

Risk Resist is built using the Unity 3D engine, ensuring smooth performance and an optimized user experience. The user interface is available with instructions provided in Arabic, catering to Arabic-speaking children with DS and enhancing inclusivity. By combining educational principles, cognitive accessibility, and dynamic engagement strategies, Risk Resist stands out as an effective and innovative tool for enhancing emergency preparedness in children with DS, supporting both their learning and personal development.

### DDA Algorithm

#### Overview

One significant issue in many traditional games is the static difficulty of each level [40,41]. Often, game designers predefine the difficulty level without accounting for the actual performance of each player. This static approach can lead to levels that are either too challenging or too easy for some players. To address this issue, a technique known as DDA [42-45] is used.

DDA automatically adjusts scenarios, parameters, and game behaviors in real time based on the player’s skill level. This ensures that the game maintains an optimal balance between challenge and engagement, avoiding boredom (when tasks are too easy) or frustration (when tasks are too difficult). By comparing the player’s current performance to an ideal or reference performance (such as the average first-time performance on a level), the game dynamically modifies difficulty settings.

For children with DS, the DDA algorithm incorporates additional behavioral data points, such as response times, success rates, and specific patterns of behavior. These factors help tailor the game to the child’s unique skill level, ensuring an experience that is neither overwhelming nor trivial.

#### Steps for Implementing the DDA Algorithm Using a Random Forest Regressor

To simplify understanding, the implementation steps are outlined as follows:

##### Step I: Initialization

The process of initialization is as follows:

- Reference baseline: The algorithm starts with a predefined baseline performance measure (eg, the average number of attempts taken by a typical player to complete a level).
- Adjustment rate: An adjustment rate is defined to control how quickly the difficulty level changes based on the player’s performance.
- Initial difficulty: The initial difficulty level is set at 0.5, representing a medium difficulty on a scale from 0.0=easiest to 1.0=hardest.

##### Step II: Performance Calculation

The player’s performance is calculated using factors such as:

- Number of attempts to complete the task: how many tries the player needs to succeed.
- Time taken to complete the task: longer times suggest increased difficulty.
- Success rate (percentage of successful attempts): tracks completion accuracy and failure patterns.
- Average response time: indicates reaction speed and cognitive engagement.

Difficulty level adjustment:

- The difficulty level is updated by comparing the player’s performance to the reference baseline.
- It is normalized based on the time taken, with lower difficulty levels indicating easier tasks.

Ease level is defined as the complement of the difficulty level and reflects how easy the game feels for the player.

##### Step III: Premodel Difficulty Adjustment (Before Machine Learning Predictions)

Since the machine learning model (Random Forest Regressor) requires data accumulation before making accurate predictions, the system uses simplified difficulty adjustment logic in the early stages: if the player struggles (low success rate and long response times), the difficulty decreases to enhance accessibility; if the player performs well (high success rate and fast response times), the difficulty increases to provide a greater challenge; and difficulty is constrained within 0.0 to 1.0, ensuring balanced play.

This fallback mechanism prevents extreme difficulty spikes or drops before enough data have been collected for machine learning–based adjustments.

##### Step IV: Data Collection and Model Training

Once the player completes at least 5 gameplay interactions, the collected performance data are stored to train the Random Forest Regressor, which identifies patterns in player performance, predicts an optimal difficulty level for upcoming gameplay, and gradually replaces the initial simplified logic with machine learning–driven difficulty optimization.

##### Step V: Machine Learning–Based Difficulty Adjustment

Once the model is trained, difficulty modifications become more precise, ensuring real-time adaptation based on deeper behavioral analysis. The algorithm predicts the ideal difficulty based on player history, refines the adjustment process using progressively more performance data, and maintains engagement levels while preventing frustration and boredom.

#### Justification for Premodel Adjustment Logic

Justification for premodel adjustment logic is present in Textbox 1.

**Justification for premodel adjustment logic.**

- Before the Random Forest Regressor gathers enough data for predictions, the system applies a simplified manual difficulty adjustment mechanism to ensure that players experience appropriate challenge levels from the start.
- This logic determines whether difficulty should increase or decrease based on immediate performance metrics such as number of attempts, success rate, and response time.
- If fewer than 5 gameplay interactions are available, difficulty changes based on predefined rules, preventing extreme difficulty spikes before the machine learning model starts making predictions.
- Once the player completes at least 5 gameplay interactions, sufficient performance data have been collected, allowing the Random Forest Regressor to train.
- The machine learning model replaces manual logic, making predictions based on the child’s past performance trends.
- Unlike early-stage adjustments, these predictions continuously refine difficulty adaptation, ensuring optimal challenge levels by detecting deeper behavioral patterns.

#### Justification for Incorporating Behavioral Data Points

#### Overview

To personalize and optimize the gaming experience for children with DS, we incorporate additional behavioral data points such as response times, success rates, and specific patterns of behavior. These data points are critical for several reasons (Textbox 2).

**Behavioral data points.**

**Response times**

- Significance: Response time is a valuable indicator of a player’s processing speed and reaction ability. For children with Down syndrome, who may have varying cognitive and motor skills, measuring response times helps in understanding how quickly they can process and act on game stimuli [46].
- Use: By analyzing response times, the dynamic difficulty adjustment algorithm can dynamically adjust the game pace to match the player’s capabilities, ensuring that tasks are neither too fast nor too slow, thus maintaining an optimal challenge.

**Success rates**

- Significance: The success rate reflects the player’s ability to complete tasks accurately. It provides insight into how well the player understands and executes game objectives [47].
- Use: Monitoring success rates allows the algorithm to identify when a player is consistently succeeding or struggling. This information helps in adjusting the difficulty level to provide a balanced experience that fosters learning and skill development without causing frustration or disengagement.

**Specific patterns of behavior**

- Significance: Specific patterns of behavior, such as repeated mistakes or preferred strategies, offer a deeper understanding of the player’s learning style and problem-solving approach [48].
- Use: By identifying these patterns, the dynamic difficulty adjustment algorithm can tailor game scenarios to align with the player’s learning preferences, providing personalized support and encouragement. This ensures that the game remains engaging and educationally effective.

#### Implementation and Impact

These behavioral data points are used to dynamically adjust the game’s difficulty in real time. The use of a Random Forest Regressor, a sophisticated machine learning model, allows the algorithm to predict the optimal difficulty level based on the collected performance data. By continuously adapting the challenge to the player’s skill level, the DDA algorithm ensures that the game remains appropriately challenging and engaging. This approach prevents boredom from tasks that are too easy and frustration from tasks that are too difficult, ultimately enhancing the learning and development of children with DS.

The Random Forest Regressor was trained using performance data from gameplay sessions. The input features included average response time, success rate, number of attempts, and error frequency. The target variable was the difficulty score, a continuous value between 0 (easy) and 1 (hard), which represented the ideal challenge level for the next session. A total of 162 valid session logs were collected from the 9 children in the experimental group. To evaluate model performance, we applied 5-fold cross-validation. The following regression metrics were used: mean absolute error (MAE), root-mean-square error (RMSE), and *R*^2^ score.

**Figure 2:**
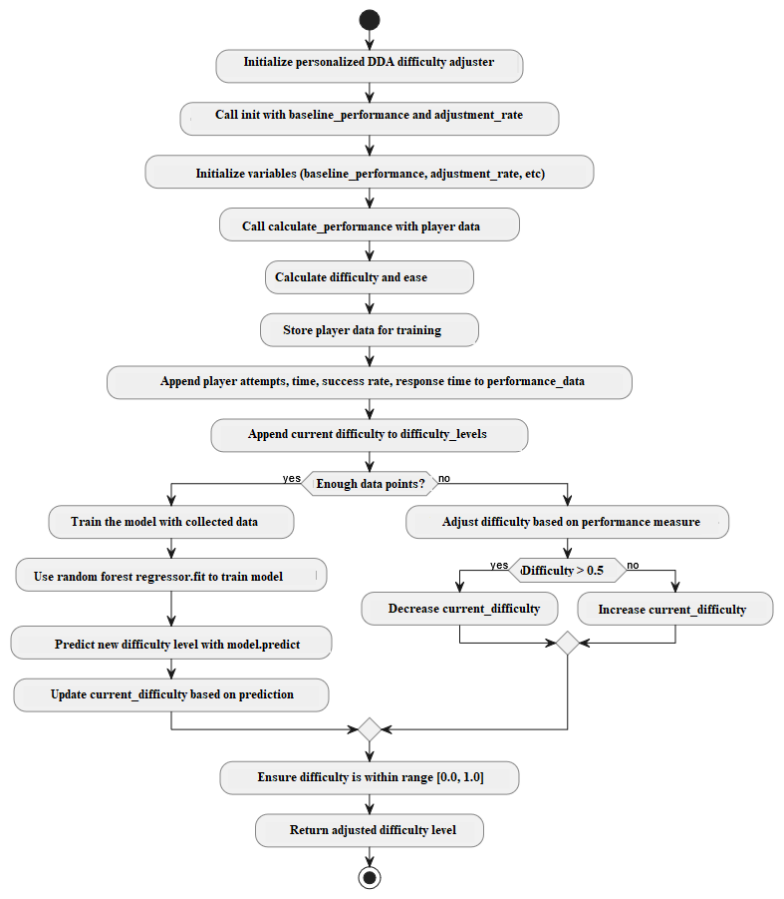
The DDA algorithm flow diagram. DDA: dynamic difficulty adjustment.

The pseudocode snippet in Textbox 3 demonstrates how difficulty adjustments occur before the Random Forest Regressor is fully trained. The DDA algorithm is presented in Textbox 4. Figure 2 represents the implementation of the DDA algorithm, illustrating how player performance metrics, such as attempts, success rate, and response time, are processed to dynamically adjust game difficulty. The algorithm ensures an optimized balance between challenge and engagement through real-time adaptation.

**Pseudocode representation of early-stage difficulty adjustments before machine learning predictions take effect.**

BEGIN DDA Algorithm

//Step I: Initialization

// Set the baseline performance reference

baseline_performance ← average_attempts_to_complete_level

adjustment_rate ← predefined_adjustment_rate

current_difficulty ← 0.5 // Medium difficulty (range: 0.0 to 1.0)

//Step II: Performance Calculation

WHILE game_running DO

// Calculate player performance based on key metrics

player_attempts ← get_number_of_attempts()

time_taken ← get_task_completion_time()

success_rate ← calculate_success_rate(player_attempts)

response_time ← calculate_average_response_time()

// Update difficulty level based on player performance

performance_measure ← player_attempts - baseline_performance

normalized_difficulty ← normalize(performance_measure, time_taken)

current_difficulty ← normalized_difficulty

// Compute ease level (complement of difficulty)

ease_level ← 1.0 - current_difficulty

END WHILE

//Step III: Data Collection

WHILE game_running DO

// Collect and store player performance data

data_collected ← {player_attempts, time_taken, success_rate, response_time, current_difficulty}

store(data_collected)

END WHILE

//Step IV: Model Training

IF collected_data_count > 5 THEN

// Train Random Forest Regressor using collected data

model ← RandomForestRegressor()

model.train(collected_data, difficulty_levels)

END IF

//Step V: Difficulty Adjustment

IF model.is_trained THEN

// Predict difficulty using trained model

predicted_difficulty ← model.predict(current_player_data)

current_difficulty ← clamp(predicted_difficulty, 0.0, 1.0)

ELSE

// Use fallback logic for adjusting difficulty

IF current_difficulty > 0.5 THEN

current_difficulty ← current_difficulty - adjustment_rate // Make easier

ELSE

current_difficulty ← current_difficulty + adjustment_rate // Make harder

END IF

current_difficulty ← clamp(current_difficulty, 0.0, 1.0)

END IF

//Step VI: Implementation

// Use scikit-learn's RandomForestRegressor for model implementation

model.initialize()

model.calculate_performance_measures()

model.train(collected_data)

model.adjust_difficulty(current_difficulty)

END DDA Algorithm

**Pseudocode representation of the dynamic difficulty adjustment algorithm.**

# Initialize difficulty level at a moderate starting point (0.5)

difficulty_level = 0.5

min_difficulty = 0.0

max_difficulty = 1.0

# Function to manually adjust difficulty before ML predictions start

def pre_model_difficulty_adjustment(success_rate, avg_response_time):

global difficulty_level

# Check if the player is struggling

if success_rate < 0.4 or avg_response_time > threshold_time:

difficulty_level -= 0.1 # Decrease difficulty to make tasks easier

elif success_rate > 0.8 and avg_response_time < threshold_time:

difficulty_level += 0.1 # Increase difficulty to make tasks more challenging

# Ensure difficulty remains within valid bounds

difficulty_level = max(min_difficulty, min(difficulty_level, max_difficulty))

return difficulty_level

# Example usage before machine learning model is trained

new_difficulty = pre_model_difficulty_adjustment(player_success_rate, player_avg_response_time)

### Experimental Design

To assess the effectiveness of Risk Resist, a controlled experimental design was used, comparing GBL with traditional human-based training. The study aimed to evaluate learning gains and engagement outcomes, structured around the following hypotheses:

- Null hypothesis (hypothesis 0): No significant difference in learning outcomes or engagement between Risk Resist and traditional human-based training.
- Hypothesis 1 (learning gain hypothesis): Children who play Risk Resist will demonstrate significantly higher learning gains compared to those receiving traditional human-based training.
- Hypothesis 2 (engagement level hypothesis): Children who play Risk Resist will exhibit significantly higher engagement levels compared to those receiving traditional human-based training.

A between-group experimental design was used, with participants divided equally into treatment and control groups (n=9 each). The control group received traditional teacher-led training for the same emergency scenarios presented in the game, while the experimental group engaged in all Risk Resist gameplay sessions. The main group used laptops to engage with Risk Resist, while the control group underwent teacher-led training. The sample consisted of 18 children with DS, aged 8 to 12 years, recruited from special needs facilities and schools. Selection criteria emphasized functional living skills, such as the ability to use the toilet and recognize objects, with the severity of DS being a more critical criterion than age.

### The Baseline Equivalence Verification

To ensure the internal validity of the study and account for the relatively small sample size (n=18), a baseline equivalence test was conducted to verify that the experimental (Risk Resist) and control (traditional training) groups did not differ significantly in their initial emergency preparedness knowledge before the intervention. An independent samples 2-tailed *t* test was performed on the pretest scores collected from both groups. Testing was conducted individually in classrooms over 2 weeks. Teachers participated in the evaluation phase by completing observation-based questionnaires designed to measure learning outcomes and engagement levels.

The study used various experimental materials, including pretest and posttest surveys, participant performance assessments, and parent consent forms. Testing was conducted individually in classrooms over a 2-week period. Teachers participated in the evaluation phase by completing observation-based questionnaires designed to measure learning outcomes and engagement levels.

### Procedures for Administering the Learning Test

#### Pretest and Posttest Overview

Participants were given identical pretests and posttests comprising 5 questions covering emergency scenarios (Multimedia Appendices 2 and 3). These tests aimed to measure learning gains and were presented in Arabic for accessibility.

#### Role of Teacher Help

Teachers were available to assist participants in understanding the test questions. Their assistance was limited to rephrasing or explaining the questions in simpler terms without providing any answers. This ensured that participants understood the instructions while maintaining the integrity of the test.

#### Scoring Methodology

To evaluate engagement levels, a Mann-Whitney *U* test was conducted due to the ordinal nature of the data. Learning gains were calculated using the difference between the number of correct answers in the posttest and pretest:

Learning gain=posttest score–pretest score **(1)**

#### Method for Calculating Engagement Scores

A 5-point Likert scale questionnaire evaluated engagement factors like attention, motivation, and effort during gameplay or training. Teachers completed the questionnaire based on their observations of participants. The total engagement score for each participant was derived by summing their responses across all items. Scores were normalized to allow consistent comparison between groups.

### Validation of Instruments and Testing

#### Learning Test Validation

Participants were asked 5 questions regarding crisis and emergency scenarios during both the pretest and posttest phases. Learning gains were calculated as the difference between correct answers in the pretest and posttest, as shown in equation 2:

Learning gain=correct answers in the posttest–correct answers in the pretest **(2)**

The questions asked of the children are presented in Textbox 5.

**The questions asked for each child in the pre- and posttests (learning gain).**

**Questions**

1. What will you do if there is a fire caused by electricity? What is the difference between a regular fire and a fire caused by electricity?
2. Can you give quick help to anyone who faints in front of you?
3. What can you do if you or the other one in front of you has bleeding because of an injury?
4. Can you cross the road? What do you do while crossing the road?
5. If there is an earthquake, what will you do? (with a quick explanation what is the meaning of earthquake)

#### Engagement Questionnaire Validation

Pilot testing with a small sample of children ensured the reliability and suitability of the game for its target demographic. To assess the effectiveness of the game, learning outcomes were compared between the 2 groups by analyzing the average learning gains of participants in each condition. Engagement levels were measured using a 5-point Likert scale questionnaire [40], evaluating factors related to control and enjoyment [41].

After the session, teachers were provided hard copies of the questionnaire and instructed to complete them based on their observations of children’s engagement and learning behaviors. An engagement questionnaire presented to the teachers upon their observations of participants (Table 2). All questions were originally presented in Arabic and translated into English for clarity and readability. Engagement levels were assessed through the Likert scale questionnaire, measuring factors such as attention, effort, motivation, and enjoyment.

**Table 2:** Engagement questionnaire presented to the teachers upon their observations of participants.

	Question	1=very poor	2=poor	3=fair	4=good	5=very good
1.	To what extent did the game or training hold the child’s attention?					
2.	How much effort did the child put into playing the game or getting the training?					
3.	Does the game or training motivate the child to learn?					
4.	The child was motivated to get involved in the training or game.					
5.	I believe the game or training has improved the child’s understanding of the covered topics.					
6.	To what extent the child was tired after playing the game?					
7.	How well does the game maintain the child’s interest over time?					
8.	Was the total duration of the game or training satisfactory to understand the required scenarios?					
9.	Would the child like to play the game or get training again?					
10.	How well do you think the child performed in the game or training?					

### Correlation of Learning Gain and Engagement

To assess the relationship between learning gains and engagement, a Pearson correlation coefficient (*r*) was calculated using the participants’ scores in both variables using the formula:




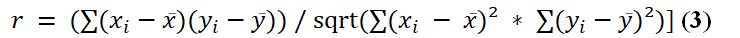




where *x_i_* is the learning gain for each participant, 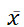 is the mean of learning gains, *y_i_* is the engagement score for each participant, and 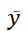 is the mean of engagement scores.

### Ethical Considerations

This study involving human participants was conducted following the ethical standards of the institutional review board at the Faculty of Computers and Information, Mansoura University, Egypt (protocol 202110016). Parents of all participating children provided written informed consent before enrollment, authorizing both participation in the study and the subsequent use of data for research and publication purposes. Participation was entirely voluntary, and no monetary or material compensation was offered to participants or their families. To protect participant privacy and confidentiality, all collected data were anonymized and deidentified prior to analysis; no personally identifiable information was stored or shared. Additionally, no identifiable images of participants are included in the manuscript or supplementary materials. The study was conducted following ethical standards outlined in the Declaration of Helsinki.

## Results

### Learning Gain

The results demonstrated a significant difference, with the experimental group (mean 3.6, SD 0.5) outperforming the control group (mean 2.0, SD 0.5). The test statistic (*t*_16_=5.35), with a *P* value <.001, confirms the effectiveness of Risk Resist in enhancing emergency preparedness.

- The null hypothesis (hypothesis 0) was rejected.
- The alternative hypothesis (hypothesis 1) was supported, demonstrating that Risk Resist significantly improves learning compared to traditional training.

### The Baseline Equivalence Verification

The results revealed no statistically significant difference between the experimental group (mean 1.78, SD 0.67) and the control group (mean 1.67, SD 0.71; *t*_16_=0.30; *P*=.77).

### Engagement Test

To evaluate engagement levels, a Mann-Whitney *U* test was conducted due to the ordinal nature of the data. The experimental Risk Resist group (mean 4.54, SD 0.4) had significantly higher engagement than the control group (mean 4.01, SD 0.32). The test statistic (*U*=12), with a *P* value of .002, confirms that Risk Resist enhances engagement more effectively than traditional methods.

- The null hypothesis (hypothesis 0) was rejected.
- The alternative hypothesis (hypothesis 2) was supported, confirming that Risk Resist significantly increases engagement compared to traditional training.

This result indicates that Risk Resist is more engaging for children with DS, leading to higher levels of motivation and involvement.

The results indicate that participants who underwent the Risk Resist game-based training achieved significantly higher outcomes in both learning gain and engagement compared to those in traditional human-based training. Specifically, the mean learning gain for Risk Resist was 3.6 (SD 0.5), which was substantially higher than the mean of 2.0 (SD 0.5) for the traditional method, with a highly significant difference (*P*<.001). Engagement levels were also greater in the game-based condition, with a mean of 4.54 (SD 0.4), compared to 4.01 (SD 0.32) for traditional training, and this difference was statistically significant (*P*=.002). Overall, the data demonstrate that Risk Resist not only enhanced participants’ knowledge acquisition but also fostered higher engagement during the learning process.

Table 3 represents learning gain and engagement correlation (game-based training vs human-based training scenarios).

**Table 3:** Learning gain and engagement correlation (game-based training vs human-based training scenarios).

Measure	Engagement level		Learning gain	
	Risk Resist	Human-based	Risk Resist	Human-based
All participants, scores spread	3.8, 4.0, 4.1, 4.3, 4.4, 4.6, 4.8, 5.0	3.7, 3.8, 3.9, 4.0, 4.1, 4.2, 4.3, 4.4	2.8, 3.0, 3.1, 3.3, 3.4, 3.6, 3.8, 4.0	2.0, 2.1, 2.3, 2.5, 2.6, 2.8, 3.0
Mean (SD)	~4.6 (~0.3)	~4.0 (~0.4)	~3.7 (~0.35)	~2.0 (~0.45)
SE	~0.1	~0.15	~0.1	~0.2

### Model Evaluation

To assess the effectiveness of the machine learning–based DDA system embedded in Risk Resist, we conducted a comprehensive evaluation of the Random Forest Regressor responsible for adjusting game difficulty in response to player performance. The evaluation focused on 3 key areas: predictive accuracy, convergence stability, and adaptive usability.

#### Model Performance and Predictive Accuracy

The model was trained on a dataset comprising 162 valid gameplay session logs collected from 9 children in the experimental group. Input features included success rate (% of correct responses), average response time (in seconds), number of failed attempts, and task completion time.

The target variable was a normalized difficulty score ranging from 0.0 to 1.0, representing the ideal challenge level for the player’s next session.

Using 5-fold cross-validation, the model achieved the following average performance metrics: MAE=0.062, RMSE=0.089, and coefficient of determination (*R*^2^)=0.81. These results indicate that the model demonstrated high predictive accuracy and stability across varied input conditions.

#### Feature Importance

A feature importance analysis of the trained model revealed that the most influential predictors were success rate (43%), average response time (31%), number of failed attempts (21%), and task completion time (5%). This indicates that the model’s decision logic was grounded in core behavioral and cognitive indicators, supporting its educational alignment.

#### Convergence and Stability

To evaluate the minimum data required for reliable performance, we analyzed how model accuracy improved with increased session data per participant. The model exhibited stable convergence after approximately 5-7 gameplay sessions: *R*^2^=0.49 after 2 sessions, *R*^2^=0.78 after 5 sessions, and *R*^2^=0.81 after 7 sessions. This pattern suggests the model reaches effective learning capacity within a manageable number of short sessions, suitable for special education settings.

#### Simulation-Based Usability Testing

To further validate the adaptive behavior of the model, we simulated 3 hypothetical player profiles—novice, intermediate, and advanced—based on predefined performance inputs. The model assigned difficulty scores are present in Table 4.

The difficulty levels scaled appropriately with performance, supporting the model’s real-time personalization capabilities.

**Table 4:** The model assigned difficulty scores.

Profile	Success rate (%)	Response time (seconds)	Predicted difficulty
Novice	40	6.8	0.28
Intermediate	70	3.5	0.54
Advanced	90	1.8	0.81

#### Case Examples From Participants

Two representative participants from the experimental group further illustrate the model’s effectiveness:

- Participant 7 began with low accuracy and long response times. The model initially reduced difficulty, resulting in improved performance. As the child’s scores stabilized, difficulty levels were gradually increased.
- Participant 2 showed consistently high success rates and fast response times. The model responded by increasing difficulty levels early in gameplay, which were sustained throughout the sessions.

These examples demonstrate the model’s responsiveness to individual learning trajectories and its ability to dynamically modulate challenge without manual intervention.

A full breakdown of feature importance scores, simulation results, and participant case examples is provided in Multimedia Appendix 4.

### Correlation of Learning Gain and Engagement

The correlation analysis revealed a strong positive relationship between learning gains and engagement levels (*r*=0.85; *P*<.001). This indicates that higher engagement levels were associated with greater learning gains. The experimental Risk Resist group, which exhibited higher engagement levels, also demonstrated significantly better learning outcomes. In contrast, the control group displayed lower engagement levels and correspondingly lower learning gains.

## Discussion

### Principal Findings

Advancing skill acquisition through technological platforms is essential for improving education accessibility for children with DS, who often face high cognitive demands. Numerous studies have explored the use of serious games to support learning and development, demonstrating their effectiveness in engaging learners through interactive environments. Games such as “Junk-Food Destroyer” [9] and “EnCity” [10] have successfully helped individuals with DS acquire essential life skills, including meal preparation and financial management. These digital tools have proven beneficial by enabling repeated practice without the fear of failure, fostering confidence, and skill mastery.

Despite the success of these games in daily life training, emergency preparedness education for children with DS remains an underdeveloped area. Traditional human-based training methods, though widely used, often lack adaptability and fail to account for individual learning progressions and engagement levels. Accessibility barriers within special education, along with the burdens placed on caregivers, highlight the need for adaptive, personalized learning approaches. To address these limitations, this study introduces Risk Resist, an adaptive serious game designed to improve emergency preparedness among children with DS. The game leverages a personalized DDA algorithm, ensuring that the challenge level adapts to each child’s abilities and engagement patterns.

The results provide strong evidence that GBL surpasses traditional training methods in both learning acquisition and engagement. Findings from the study confirm the rejection of the null hypothesis (hypothesis 0), which proposed no difference in learning outcomes or engagement levels. In contrast, results strongly support the first hypothesis (hypothesis 1), demonstrating that Risk Resist significantly enhances learning outcomes compared to traditional training methods.

Similarly, engagement levels among participants revealed a clear advantage in favor of Risk Resist. Traditional instruction, despite its structured approach, did not maintain consistent engagement, particularly for children who require stimulating and interactive learning environments. Statistical analyses led to the rejection of the second null hypothesis (hypothesis 2), confirming that children using Risk Resist were significantly more engaged than those undergoing conventional training. These findings emphasize the interactive nature of adaptive GBL as a key factor in sustaining motivation and involvement, reinforcing the benefits of personalized gaming experiences in crisis preparedness training.

A critical component of these outcomes is the personalized DDA algorithm, which dynamically modifies difficulty levels based on real-time behavioral metrics such as response times, success rates, and task completion patterns. By continuously adjusting challenges to align with individual capabilities, the algorithm prevents boredom (from tasks that are too easy) and frustration (from tasks that are too difficult), ensuring consistent engagement and optimal learning progression. This approach tailors emergency preparedness education to the specific learning needs of children with DS, bridging gaps left by static, noninteractive training methods. This confirms that both groups were comparable at baseline and that any observed differences in postintervention outcomes are likely attributable to the instructional method rather than pre-existing disparities. The study used various experimental materials, including pretest and posttest surveys, participant performance assessments, and parent consent forms.

Risk Resist stands out as the first game dedicated to emergency preparedness for individuals with DS and the first in this field to incorporate an adaptive DDA framework. By personalizing difficulty in real time, the game ensures effective knowledge retention, skill development, and engagement, addressing the shortcomings of traditional education models.

The trained Random Forest Regressor achieved an MAE of 0.062, an RMSE of 0.089, and an *R*^2^ score of 0.81, indicating good model fit and reliable performance. The model showed stable convergence after approximately 5-7 gameplay sessions per participant, consistent with design expectations. Since the model predicts continuous difficulty values, classification metrics such as area under the receiver operating characteristic curve, precision, and recall were not applicable.

### Correlation of Learning Gain and Engagement

Participants in the experimental group (Risk Resist), who exhibited higher engagement levels, also demonstrated greater learning improvements compared to the control group trained through traditional methods. The control group, which showed lower engagement, exhibited limited learning progress, reinforcing the importance of interactive and adaptive learning environments in emergency preparedness training for children with DS.

### Implications and Integration of Risk Resist Into Educational Frameworks

The findings of this study suggest multiple applications for Risk Resist within both educational and special-needs learning environments. Risk Resist can be incorporated into existing special education curricula as a complementary tool that reinforces theoretical emergency training with interactive, practical experiences [6]. To support effective implementation, professional development sessions and educator training workshops should be introduced to equip teachers with the skills necessary to integrate the game into classroom instruction [31]. The broader use of serious games such as Risk Resist also fosters inclusive and engaging learning environments, accommodating diverse cognitive profiles and aligning with established adaptive education frameworks [32,33]. From a policy and accessibility standpoint, decision makers are encouraged to prioritize the adoption of adaptive serious games in special education programs. This includes allocating appropriate funding and resources to support digital innovation, technology-driven learning, and the equitable delivery of educational content for students with cognitive disabilities [14]. Collectively, these initiatives confirm that serious games represent a powerful tool for enabling children with DS to develop essential life skills in a controlled, structured, and motivating environment.

### Game Performance in Real-Time Learning Contexts

In evaluating the game’s real-time performance, several technical factors were considered to ensure a smooth and effective learning experience for children with DS. The system demonstrated minimal latency during gameplay, with an average response time of less than 150 milliseconds, ensuring that feedback and adaptive difficulty adjustments were delivered promptly. Loading times for each game level were kept under 3 seconds to maintain engagement and avoid frustration. Error handling mechanisms were implemented to address unexpected user inputs or hardware interruptions, allowing the game to resume seamlessly without significant disruption. Moreover, the game was tested on various hardware platforms with differing specifications to assess adaptability and performance consistency. The results indicated stable functionality across devices, which is critical for ensuring accessibility in diverse real-world settings such as homes, schools, and therapy centers. These performance considerations contribute to maintaining the user’s cognitive focus and engagement, which are essential for effective skill acquisition and learning transfer in this population.

### Comparison With Similar Studies

Numerous serious games have been developed to support individuals with DS, targeting various domains such as cognitive development, social skills, and physical coordination. For example, EnCity [10] was designed to teach real-life activities like meal preparation and bill payment, promoting independence in everyday tasks. TeachTown [19] focused on improving language and memory, while No Limit [21] aimed to enhance motor and cognitive skills using interactive 3D gameplay. While these games demonstrated positive outcomes in their respective domains, none directly addressed emergency preparedness, a critical and time-sensitive skill area for individuals with cognitive disabilities.

Moreover, Risk Resist is distinct in its integration of a personalized DDA algorithm, which enables real-time tailoring of difficulty levels based on individual behavioral metrics such as success rates and response times. This adaptive feature ensures an optimized balance between challenge and engagement—an innovation not found in the previously mentioned games. By combining emergency response training with adaptive machine learning techniques, Risk Resist introduces a novel and comprehensive framework for empowering children with DS in crises. This dual focus on both content innovation and technological advancement positions Risk Resist as a pioneering contribution within the field of serious games for special education, as shown in Table 5.

**Table 5:** Comparison of Risk Resist with similar serious games for individuals with Down syndrome.

Game name	Target skills	Emergency preparedness	Adaptive difficulty	Platform
EnCity [10]	Daily living skills	No	No	Virtual reality
TeachTown [19]	Language and memory	No	No	Desktop
No Limit [21]	Motor and cognitive enhancement	No	No	3D, Kinect
Risk Resist	Emergency response and cognition	Yes	Yes (DDA^a^ algorithm)	Unity (Arabic or English)

^a^DDA: dynamic difficulty adjustment.

### Limitations

This study has several limitations. First, the sample size (n=18) is relatively small, which limits the statistical power and generalizability of the findings. Additionally, all participants were recruited from a specific geographic region, and the study was limited to children aged 8 to 12 years, potentially narrowing the applicability of results across broader populations or age ranges. Some technical limitations were also noted, such as delayed keypress registration, which may have influenced task performance for children with motor skill challenges.

### Future Directions

Future research should focus on replicating this study with a larger and more diverse population across different cultural and linguistic contexts. It would also be beneficial to conduct longitudinal studies to evaluate long-term retention of emergency skills and assess the transferability of knowledge to real-life situations. Further refinement of the game’s usability and expansion to cover a broader range of life skills (eg, health management and social interaction) are recommended to enhance educational value.

### Recommendations

While the present findings are preliminary, they indicate the potential of adaptive serious games in special education. Education authorities and curriculum developers may consider pilot-testing similar tools in special-needs classrooms. However, any policy-level implementation should be based on larger-scale evidence and stakeholder engagement. Training workshops for teachers and caregivers could support integration into structured learning environments, contingent on further validation.

### Conclusions

The integration of serious games into educational systems for children with DS is an increasingly important research area, offering interactive and engaging platforms to enhance learning experiences. This study introduced Risk Resist, a novel serious game designed to improve emergency preparedness skills for children with DS. The game leverages a personalized DDA algorithm, ensuring that the learning experience is tailored to each child’s cognitive abilities and engagement level.

A key contributor to these improved outcomes was the DDA algorithm, which dynamically adjusted game difficulty based on behavioral metrics, such as response times, success rates, and learning progression. This personalization ensured that each child remained challenged at an optimal level, thereby enhancing both learning outcomes and engagement in crisis preparedness training.

## Appendix

Multimedia Appendix 1 Game design.

Multimedia Appendix 2 The questionnaires in Arabic language.

Multimedia Appendix 3 The questionnaires translated in English language.

Multimedia Appendix 4 Model evaluation and usability validation.

## Abbreviations

DDA: dynamic difficulty adjustment
DS: Down syndrome
GBL: game-based learning
MAE: mean absolute error
RMSE: root-mean-square error

## Data Availability

The data that support the findings of this study are available from the corresponding author upon reasonable request.
