# Supplementary material for: Empowering Children With Down Syndrome by Enhancing Emergency Preparedness Through Serious Games: Quasi-Experimental Study With a Between-Group Design
Source: JMIR Serious Games. 2025 Oct 17;13:e73690. doi: 10.2196/73690 (PMC12579303; doi:10.2196/73690)
Supplement: Multimedia Appendix 1 [file games_v13i1e73690_app1.docx]

### **Prototype Creation & Iterative Design**

- **Hand modeling**: Initial sketches of characters, environments, and gameplay mechanics (Figure S1)
- **Graphical User Interface (GUI) Design**: Developing intuitive controls, accessible menus, and interactive feedback mechanisms (Figure S2)
- Early prototypes were tested for usability and comprehension, and refined based on feedback from educators and experts.

**
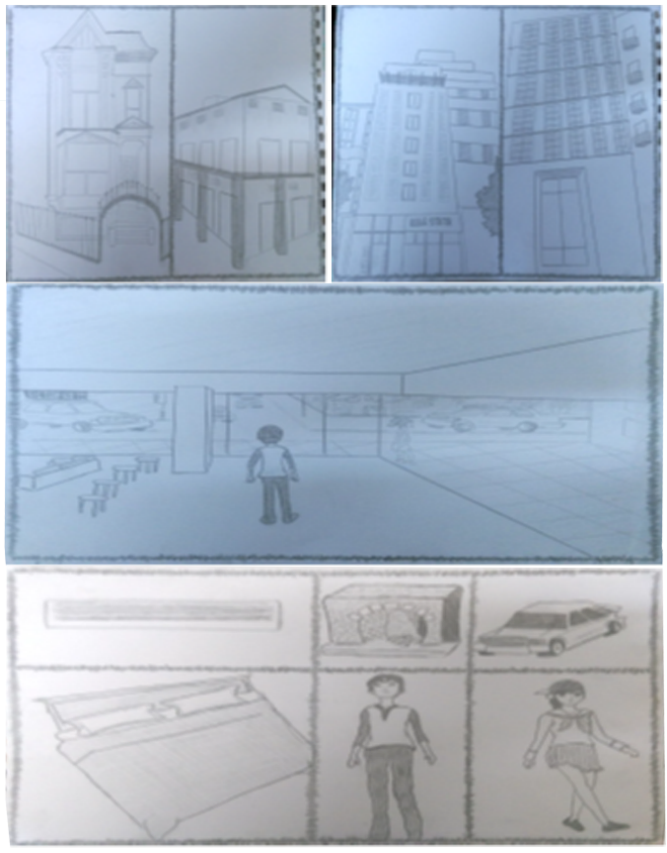
**

**Figure S1.** Hand modeling for “Risk Resist”


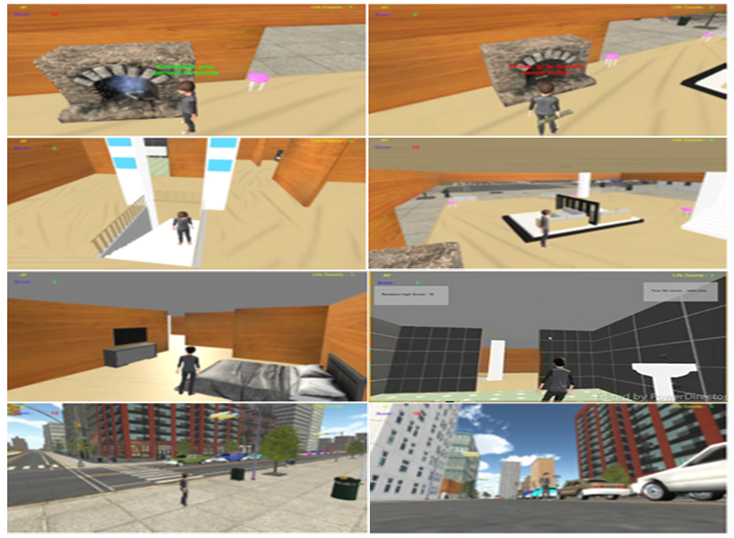


**Figure S2.** GUI Design for “Risk Resist” (Real screenshots from the game)
