# Supplementary material for: Empowering Children With Down Syndrome by Enhancing Emergency Preparedness Through Serious Games: Quasi-Experimental Study With a Between-Group Design
Source: JMIR Serious Games. 2025 Oct 17;13:e73690. doi: 10.2196/73690 (PMC12579303; doi:10.2196/73690)
Supplement: Multimedia Appendix 2 [file games_v13i1e73690_app2.docx]

**استبيان تقييم التعلم (قبل وبعد)**

| **رقم السؤال** | **السؤال باللغة العربية** |
| --- | --- |
| 1 | ماذا ستفعل إذا اندلع حريق بسبب الكهرباء؟ ما الفرق بين الحريق العادي والحريق الناتج عن الكهرباء؟ |
| 2 | هل يمكنك تقديم مساعدة سريعة لشخص أغمي عليه أمامك؟ |
| 3 | ماذا يمكنك أن تفعل إذا تعرضت أنت أو أي شخص أمامك لنزيف بسبب إصابة؟ |
| 4 | هل يمكنك عبور الطريق؟ ماذا تفعل أثناء عبور الطريق؟ |
| 5 | إذا حدث زلزال، ماذا ستفعل؟ (اشرح بسرعة ما معنى الزلزال) |
