# Supplementary material for: Empowering Children With Down Syndrome by Enhancing Emergency Preparedness Through Serious Games: Quasi-Experimental Study With a Between-Group Design
Source: JMIR Serious Games. 2025 Oct 17;13:e73690. doi: 10.2196/73690 (PMC12579303; doi:10.2196/73690)
Supplement: Multimedia Appendix 3 [file games_v13i1e73690_app3.docx]

**Learning Evaluation Questionnaire (Pre and Post)**

| **Question No.** | **Question Translated From Arabic** |
| --- | --- |
| 1 | What will you do if a fire breaks out due to electricity? What is the difference between a regular fire and an electrically caused fire? |
| 2 | Can you provide quick help to someone who has fainted in front of you? |
| 3 | What can you do if you or someone in front of you is bleeding due to an injury? |
| 4 | Can you cross the road? What do you do while crossing the road? |
| 5 | If an earthquake occurs, what will you do? (Quickly explain what an earthquake means) |
