# Supplementary material for: Empowering Children With Down Syndrome by Enhancing Emergency Preparedness Through Serious Games: Quasi-Experimental Study With a Between-Group Design
Source: JMIR Serious Games. 2025 Oct 17;13:e73690. doi: 10.2196/73690 (PMC12579303; doi:10.2196/73690)
Supplement: Multimedia Appendix 4 [file games_v13i1e73690_app4.docx]

This appendix presents a comprehensive evaluation of the **Random Forest Regressor** utilized within the *Risk Resist* serious game to implement **Dynamic Difficulty Adjustment (DDA)**. The goal is to demonstrate the model’s reliability, adaptability, and alignment with individual player performance through quantitative and simulation-based analyses.

**1. Feature Importance Analysis**

The model was trained using key performance indicators collected during gameplay, including:

- **Success rate (% of correct task completions)**
- **Average response time (in seconds)**
- **Number of failed attempts**
- **Task completion time**

Following model training, feature importance was extracted to assess the contribution of each input variable to difficulty prediction.

| **Feature** | **Importance Score** |
| --- | --- |
| Success rate | 0.43 |
| Average response time | 0.31 |
| Number of failed attempts | 0.21 |
| Task completion time | 0.05 |

*Interpretation:* The model primarily relies on success and speed-based performance signals, confirming its alignment with behavioral adaptation principles.

**2. Model Performance Metrics**

To evaluate the predictive quality of the model, **5-fold cross-validation** was conducted using 162 session logs collected from the experimental group.

| **Metric** | **Value** |
| --- | --- |
| Mean Absolute Error (MAE) | 0.062 |
| Root Mean Squared Error (RMSE) | 0.089 |
| Coefficient of Determination (R²) | 0.81 |

These results indicate strong predictive accuracy and generalizability across gameplay data.

**3. Convergence Analysis**

To assess the model’s learning stability, performance metrics were analyzed based on the number of gameplay sessions per participant.

| **Number of Sessions** | **R² Score** |
| --- | --- |
| 2 | 0.49 |
| 3 | 0.66 |
| 5 | 0.78 |
| ≥7 | 0.81 |

*Conclusion:* The model achieved performance convergence after 5–7 sessions, which is suitable for short-term, iterative educational applications in special needs contexts.

**4. Simulation-Based Validation**

To evaluate the model’s adaptability, three simulated player profiles were created based on hypothetical performance metrics.

| **Profile** | **Average Success Rate** | **Average Response Time (s)** | **Predicted Difficulty Score** |
| --- | --- | --- | --- |
| Novice | 40% | 6.8 | 0.28 |
| Intermediate | 70% | 3.5 | 0.54 |
| Advanced | 90% | 1.8 | 0.81 |

*Observation:* The DDA model appropriately adjusted difficulty levels according to the simulated player’s ability, confirming its behavioral responsiveness and calibration.

**5. Representative Participant Case Studies**

**Participant 7 – Low Initial Performance**

- **Session 1**: Success rate = 33%, response time = 7.2s
- **Session 2**: Difficulty score reduced by 0.2 → Success rate increased to 60%
- **Session 4**: Model began increasing difficulty gradually based on improved accuracy and reduced response times

**Participant 2 – Consistently High Performance**

- **Session 1**: Success rate = 89%, response time = 2.1s
- **By Session 3**: Difficulty score increased from 0.55 to 0.80
- Performance remained high, with sustained engagement throughout sessions

These cases illustrate the model’s effective real-time personalization and ability to maintain a balanced level of challenge tailored to each learner.
